# Circular RNA circMRPS35 represses malignant progression in osteosarcoma cells via targeting miR-105-5p/FOXO1

**DOI:** 10.18632/aging.206022

**Published:** 2024-08-05

**Authors:** Chunshan Jiang, Zhe Jiang, Xuewu Zhang

**Affiliations:** 1 Department of Immunology, College of Medicine, Yanbian University, Yanji, Jilin 133002, P.R. China; 2 Department of Biochemistry and Molecular Biology, College of Medicine, Yanbian University, Yanji, Jilin 133002, P.R. China; 3 Department of Spine Surgery, Jilin Central Hospital, Jilin 132011, P.R. China

**Keywords:** osteosarcoma, progression, circMRPS35, miR-105-5p, FOXO1

## Abstract

Osteosarcoma is a highly metastatic, aggressive bone cancer that occurs in children and young adults worldwide. Circular RNAs (circRNAs) are crucial molecules for osteosarcoma progression. In this study, we aimed to investigate the impact of circMRPS35 overexpression and its interaction with FOXO1 via evaluating apoptosis, cell cycle, and bioinformatic analyses on the malignant development of osteosarcoma in MG63 and MNNG/HOS cells. We found that circMRPS35 overexpression reduced osteosarcoma cell viability and inhibited tumor growth *in vivo*. It increased the apoptosis rate and induced cell cycle arrest in osteosarcoma cells. We identified a potential interaction between circMRPS35 and FOXO1 with miR-105-5p using bioinformatics analysis. Overexpression of circMRPS35 decreased miR-105-5p expression, whereas miR-105-5p mimic treatment increased its expression. This mimic also suppressed the luciferase activity of circMRPS35 and FOXO1 and reduced FOXO1 expression. Overexpression of circMRPS35 elevated FOXO1 protein levels, but this effect was reversed by co-treatment with the miR-105-5p mimic. We demonstrated that inhibiting miR-105-5p decreased viability and induced apoptosis. Overexpression of FOXO1 or treatment with a miR-105-5p inhibitor could counteract the effects of circMRPS35 on viability and apoptosis in osteosarcoma cells. Therefore, we concluded that circMRPS35 suppressed the malignant progression of osteosarcoma via targeting the miR-105-5p/FOXO1 axis.

## INTRODUCTION

Osteosarcoma (OS) is an aggressive bone cancer that occurs in children and young adults worldwide [1]. Treatment primarily consists of surgical resection and radiotherapy, which can achieve a 5-year survival rate of over 70% in patients without metastatic disease [2]. However, approximately 20% of patients with advanced disease exhibit an unsatisfactory response to standard therapies [2, 3]. This underscores the urgency of developing new therapeutic strategies.

The forkhead box protein 1 (FOXO1), a transcription factor, is involved in gene regulation during cell proliferation, growth, differentiation, and other processes [4]. Post-translational modifications, such as phosphorylation by AKT and FOXO1 acetylation significantly modulate biological functions including autophagy and apoptosis [5, 6]. A previous study suggested that histone deacetylase inhibitors induced autophagy via the FOXO1 signaling pathway in OS [7].

Over the past decades, noncoding RNAs have been widely reported to play roles in regulating gene expression and cell functions, including autophagy and OS progression [8, 9]. Circular RNAs (circRNAs) are a type of noncoding RNA characterized by a unique covalently closed RNA structure; their aberrant levels are closely correlated with cancer development, including that of OS [10–13]. Mechanistically, circRNAs mainly regulate gene expression via acting as competitive RNAs for microRNAs (miRNAs) or directly forming complexes with proteins [14]. For instance, circTADA2A promotes the survival, migration, and invasion of OS cells via regulating miR-203a-3p to upregulate CREB3 levels [15].

As a newly identified tumor suppressor in gastric cancer cells, circMRPS35 has been reported to directly regulate the histone modification of FOXO1 and FOXO3a via recruiting lysine acetyltransferase KAT7 [16]. In this study, we aimed to determine the role of circMRPS35 in OS progression. We found that circMRPS35 modulated the growth and apoptosis of OS cells via sponging miR-105-5p and activating FOXO1 expression. This work provides a novel therapeutic target for OS treatment.

## RESULTS

### Overexpression of circMRPS35 inhibits OS cell proliferation *in vitro* and *in vivo*

**Figure 1:**
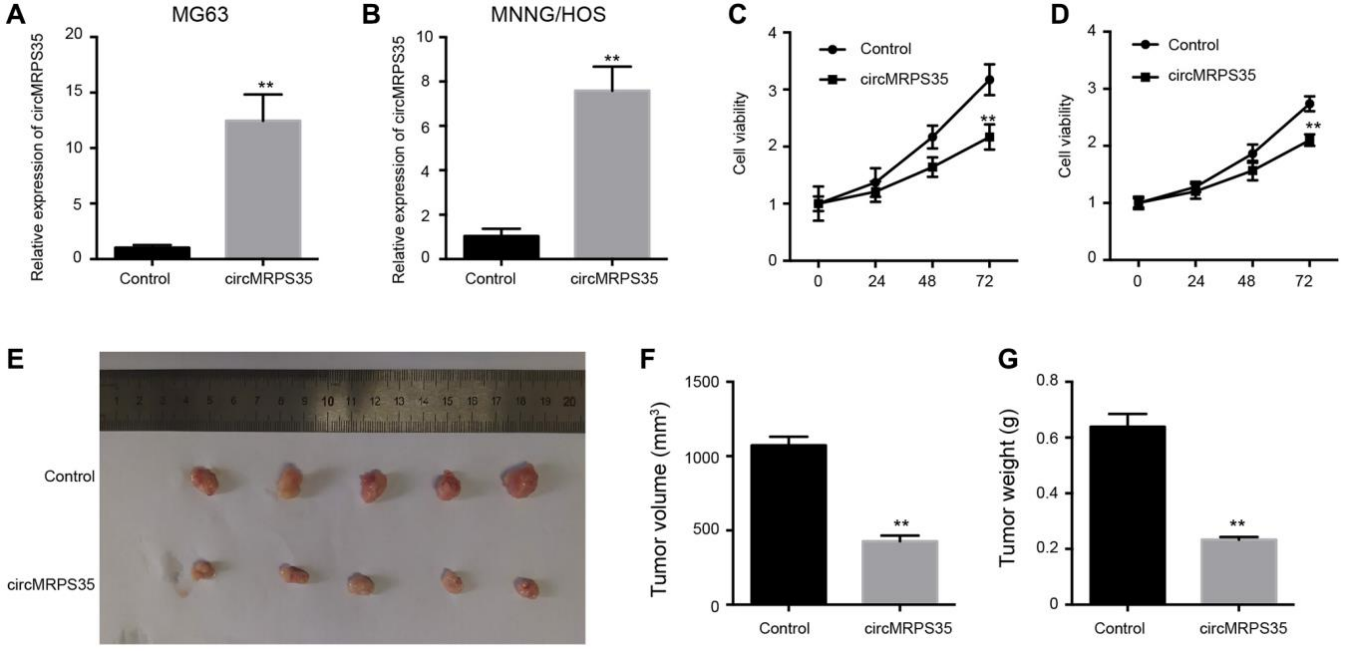
**The overexpression of circMRPS35 inhibits cell proliferation of osteosarcoma *in vitro* and *in vivo*.** (**A**–**D**) The MG63 and MNNG/HOS cells were treated with circMRPS35 overexpressing plasmid. (**A**, **B**) The expression of circMRPS35 was detected by qPCR. (**C**, **D**) The cell viability was measured by CCK-8 assay. (**E**–**G**) The tumorigenesis analysis was performed in nude mice injected with MG63 cells treated with circMRPS35 overexpressing plasmid. ^**^P < 0.01.

To investigate the effect of circMRPS35 on OS, MG63 and MNNG/HOS cells were treated with a circMRPS35 overexpression plasmid. Successful overexpression of circMRPS35 in MG63 and MNNG/HOS cells was confirmed (Figure 1A, 1B), and it was found to diminish the viability of both MG63 and MNNG/HOS cells (Figure 1C, 1D). It also significantly inhibited tumor growth in OS cells *in vivo* (Figure 1E–1G).

### CircMRPS35 contributes to apoptosis and regulates OS cell cycle

**Figure 2:**
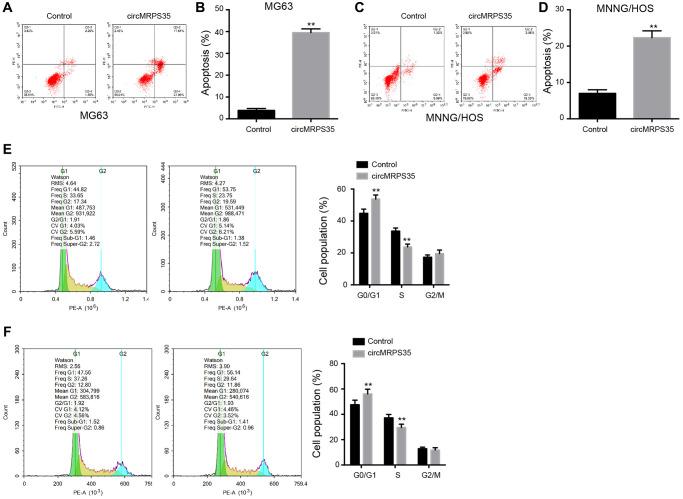
**CircMRPS35 contributes to apoptosis and regulates cell cycle of osteosarcoma cells.** (**A**–**F**) The MG63 and MNNG/HOS cells were treated with circMRPS35 overexpressing plasmid. (**A**–**D**) The cell apoptosis was analyzed by flow cytometry analysis. (**E**, **F**) The cell cycle was determined by flow cytometry analysis. ^**^P < 0.01.

We investigated the role of circMRPS35 in modulating apoptosis and cell cycle in MG63 and MNNG/HOS cells. Overexpression of circMRPS35 increased the apoptosis rate in both MG63 and MNNG/HOS cells (Figure 2A–2D). Concurrently, it also resulted in an increased proportion of MG63 and MNNG/HOS cells in the G0/G1 phase, whereas the S phase cell population was reduced (Figure 2E, 2F).

### CircMRPS35 serves as a miR-105-5p sponge in OS cells

**Figure 3:**
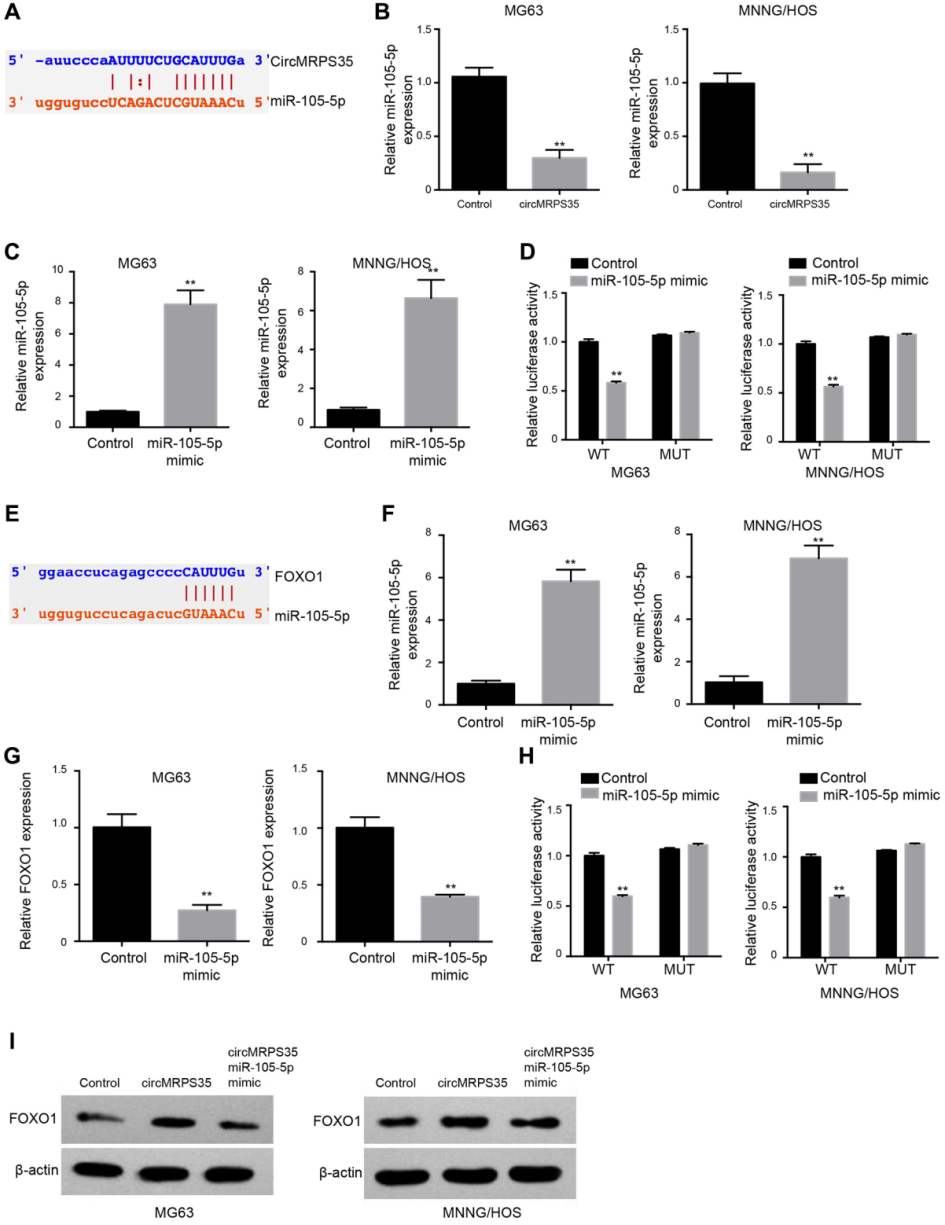
**CircMRPS35 serves as a sponge of miR-105-5p to promote FOXO1 expression in osteosarcoma cells.** (**A**) The interaction of circMRPS35 and miR-105-5p was predicted in ENCORI database. (**B**) The expression of miR-105-5p was measured by qPCR in MG63 and MNNG/HOS cells treated with circMRPS35 overexpressing plasmid. (**C**, **D**) The MG63 and MNNG/HOS cells were transfected with miR-105-5p mimic. (**C**) The expression of miR-105-5p was measured by qPCR. (**D**) The luciferase activity of circMRPS35 was analyzed by luciferase reporter gene assay. (**E**) The interaction of FOXO1 and miR-105-5p was predicted in ENCORI database. (**F**–**H**) The MG63 and MNNG/HOS cells were transfected with miR-105-5p mimic. (**F**) The expression of miR-105-5p was measured by qPCR. (**G**) The expression of FOXO1 was detected by qPCR. (**H**) The luciferase activity of FOXO1 3’UTR was analyzed by luciferase reporter gene assay. (**I**) The expression of FOXO1 was detected by Western blot analysis in MG63 and MNNG/HOS cells treated with circMRPS35 overexpressing plasmid and miR-105-5p mimic. ^**^P < 0.01.

We identified a potential interaction between circMRPS35 and miR-105-5p via bioinformatics analysis (Figure 3A). Overexpression of circMRPS35 decreased miR-105-5p expression (Figure 3B), whereas treatment with a miR-105-5p mimic increased its expression (Figure 3C). Additionally, the luciferase activity of circMRPS35 was suppressed by the miR-105-5p mimic in MG63 and MNNG/HOS cells (Figure 3D).

### miR-105-5p targets FOXO1 in OS cells

Subsequently, we explored the potential interaction between FOXO1 and miR-105-5p via bioinformatics analysis (Figure 3E). Treatment with miR-105-5p mimic induced miR-105-5p (Figure 3F) expression, whereas it repressed FOXO1 expression (Figure 3G). In MG63 and MNNG/HOS cells, the luciferase activity associated with FOXO1 was suppressed by the miR-105-5p mimic (Figure 3H). Overexpression of circMRPS35 enhanced FOXO1 protein levels, but this effect was reversed by co-treatment with the miR-105-5p mimic in both MG63 and MNNG/HOS cells (Figure 3I).

### Inhibition of miR-105-5p reduces viability and induces apoptosis of OS cells

We subsequently evaluated the role of miR-105-5p in regulating the viability and apoptosis of OS cells. We found that the miR-105-5p inhibitor reduced the viability of MG63 and MNNG/HOS cells (Supplementary Figure 1A, 1B). Additionally, apoptosis induction in MG63 and MNNG/HOS cells was promoted by the inhibitor (Supplementary Figure 1C–1F).

### OS cell viability and apoptosis are modulated by circMRPS35 via targeting miR-105-5p/FOXO1 axis

**Figure 4:**
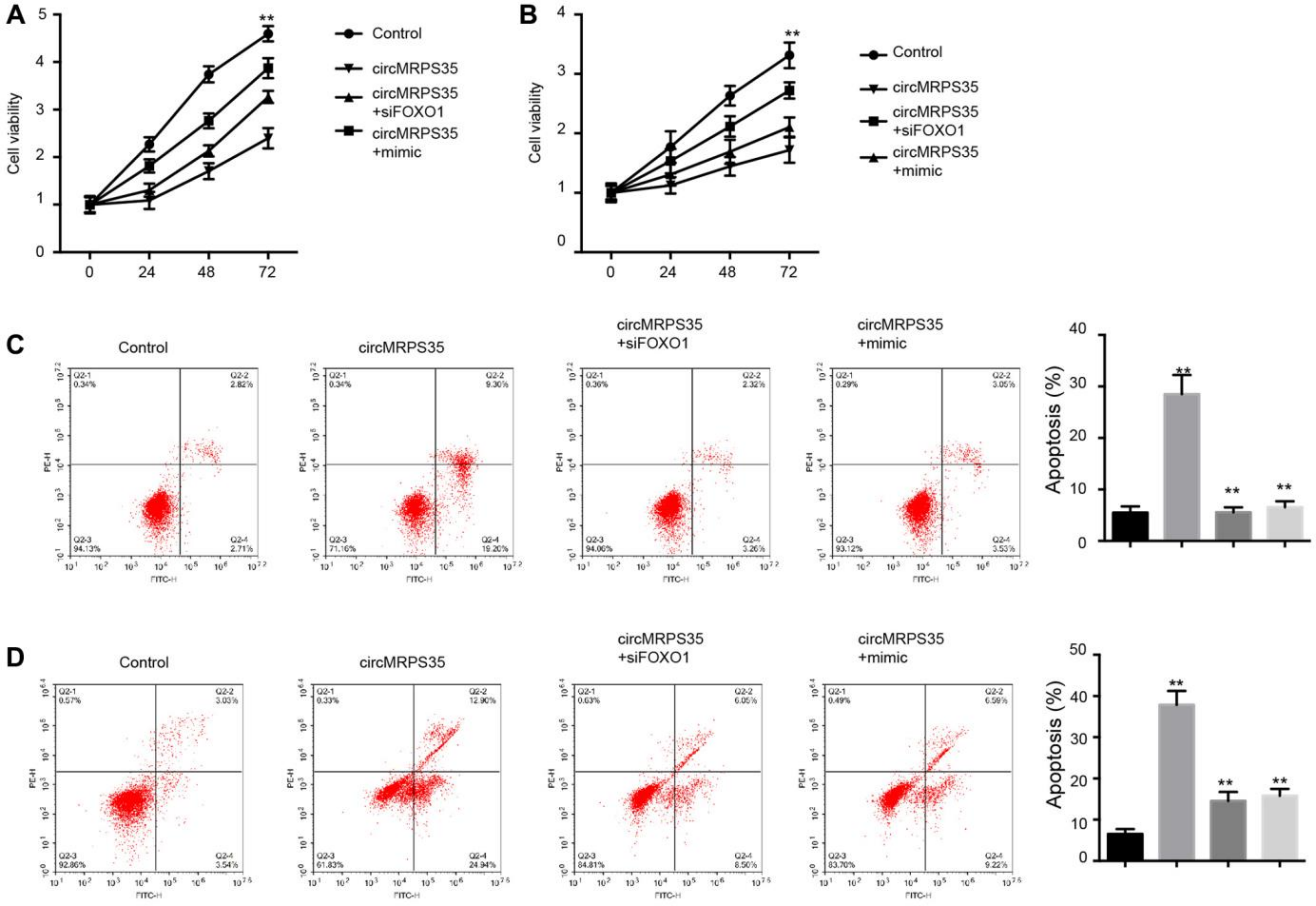
**CircMRPS35 regulates viability and apoptosis of osteosarcoma cells by targeting miR-105-5p/FOXO1 axis.** (**A**–**D**) The MG63 and MNNG/HOS cells were treated with circMRPS35 overexpressing plasmid, or co-treated with circMRPS35 overexpressing plasmid and miR-105-5p mimic or FOXO1 siRNA. (**A**, **B**) The cell viability was analyzed by CCK-8 assay. (**C**, **D**) The cell apoptosis was measured by flow cytometry analysis. ^**^P < 0.01.

We identified that circMRPS35 overexpression suppressed MG63 and MNNG/HOS cell viability; however, this effect was reversed by either FOXO1 inhibition or the miR-105-5p mimic (Figure 4A, 4B). Additionally, circMRPS35 induced apoptosis in MG63 and MNNG/HOS cells, an effect that was obstructed by FOXO1 knockdown or the miR-105-5p mimic (Figure 4C, 4D).

## DISCUSSION

OS is a highly metastatic, aggressive bone cancer that occurs in children and young adults worldwide. CircRNAs have been identified as crucial molecules in OS progression. However, the function of the circular RNA circMRPS35 in OS remains elusive. Here, we uncovered the effect of circMRPS35 on the malignant development of OS.

Multiple circRNAs have been identified as modulators of OS progression. Circular RNA hsa_circ_0005909 has been reported to regulate OS progression through the miR-936/HMGB1 signaling pathway [17]. Circular RNA circ_0000337 promotes OS via a miR-4458/BACH1 axis [18]. Circular RNA circ_0032462 contributes to OS development via enhancing KIF3B expression [19]. Circular RNA circTADA2A facilitates OS progression and metastasis through the miR-203ª-3p/CREB3 axis [15]. These findings including the effects of circMRPS35 overexpression observed in this study suggest that circMRPS35 suppresses the malignant progression of OS, providing new evidence regarding the function of circMRPS35 in this disease.

Furthermore, miRNAs play a role in modulating OS. The miRNA-188-5p has been shown to suppress OS progression via targeting CCNT2 [20]. The miRNA-744 promotes OS cell proliferation via inhibiting PTEN [21], whereas miRNA-206 decreases OS metastasis via targeting Notch3 [22]. The LncRNA SNHG3/miRNA-151a-3p/RAB22A signaling pathway modulates OS cell invasion and migration [23]. Additionally, FOXO1 has been found to inhibit oncogenesis in OS through Wnt/β-catenin signaling [24]. In our study, we identified a potential interaction between circMRPS35 and miR-105-5p, wherein circMRPS35 overexpression reduced miR-105-5p expression, while miR-105-5p mimic treatment increased its expression. A similar potential interaction was observed between FOXO1 and miR-105-5p. We confirmed that inhibiting miR-105-5p reduced viability and induced apoptosis in OS cells. Overexpression of FOXO1 or treatment with a miR-105-5p inhibitor could counteract the effects of circMRPS35 on viability and apoptosis in OS cells. The clinical correlation between circMRPS35, miR-105-5p, and FOXO1 hence warrants further investigation.

We concluded that the circular RNA circMRPS35 inhibited the malignant progression of OS via targeting the miR-105-5p/FOXO1 axis. Therefore, circMRPS35, miR-105-5p, and FOXO1 could serve as potential therapeutic targets in OS treatment.

## MATERIALS AND METHODS

### Cell lines

The human OS cell lines U2OS and MNHG/HOS were obtained from the American Type Culture Collection (ATCC, VA, USA). Cells were cultured in minimum essential medium (Hyclone, UT, USA) containing 10% fetal bovine serum (Gibco, Thermo Fisher Scientific, MA, USA) and 1% penicillin-streptomycin (Gibco) in a humidified atmosphere at 37°C with 5% carbon dioxide.

### Cell transfection

Overexpression plasmid pCMV-circMRPS35, miR-105-5p mimics and inhibitors, siRNA targeting FOXO1 (si-FOXO1), and the corresponding negative control (NC), were purchased from Gene Pharma (Shanghai, China). U2OS and MNHG/HOS cells were seeded into six-well plates for cell transfection. Lipofectamine 2000 (Invitrogen, CA, USA) was used to introduce oligonucleotides into the cells as per manufacturer’s instructions. Cells were harvested 48 h post-transfection for subsequent experiments.

### Cell counting kit 8 (CCK-8 assay)

Cell proliferation was assessed using CCK-8 (Beyotime, Shanghai, China) as per manufacturer’s instructions. Briefly, U2OS and MNHG/HOS cells (5 × 10^3^ cells/well) were seeded in 96-well plates with the specified oligonucleotides following transfection. Cells were incubated for 24, 48, and 72 h, after which 10 μl of CCK-8 reagent was added to each well and incubated for an additional hour. Absorbance at 450 nm was measured using a microplate spectrometer (Thermo Fisher Scientific).

### Cell apoptosis

The Annexin V-FITC/propidium iodide (PI) apoptosis detection kit (Beyotime) was used to assess cell apoptosis. After transfection, cells were collected, suspended in binding buffer, and stained with 5 μl Annexin V-FITC and 5 μl PI, followed by a 30-min incubation. Apoptotic cells were then immediately detected using flow cytometry (BD Biosciences, NJ, USA).

### Cell cycle

To evaluate the cell cycle, cells were collected post-transfection, fixed in 75% ethanol overnight, and subsequently incubated with PI reagent (Beyotime) for 30 min in the dark. The samples were then analyzed using a flow cytometer (BD Biosciences).

### Quantitative real-time polymerase chain reaction

TRIzol reagent (Thermo Fisher Scientific) was utilized to extract RNA from cells post-transfection, as per manufacturer’s protocol. The RNA was reverse-transcribed to cDNA using a First Strand synthesis kit (Takara Bio Inc., Shiga, Japan). The expression levels of circMRPS35, miR-105-5p, and FOXO1 were detected using SYBR Green qPCR Master Mix (Takara), employing the 2^−ΔΔCt^ method, and normalized to U6 and GAPDH, respectively.

### Western blotting

U2OS and MNHG/HOS cells were lysed with ice-cold RIPA buffer (Beyotime) to extract total proteins. The proteins were quantified using a BCA kit (Beyotime), separated using sodium dodecyl-sulfate polyacrylamide gel electrophoresis, and transferred to polyvinylidene difluoride membranes. The membranes were blocked with 5% skim milk and then incubated with specific primary antibodies against FOXO1 and GAPDH (Abcam, Cambridge, UK) overnight at 4°C. The following day, the proteins were incubated with the corresponding secondary anti-rabbit antibody (Abcam) at room temperature for 2 h. Proteins were detected using an enhanced chemiluminescence reagent (Millipore, MA, USA).

### Xenograft mice model

Male BALB/c SCID mice, aged six weeks, were obtained from Charles River Laboratories (Beijing, China). U2OS cells (2 × 10^6^ per mouse) were suspended in 50 μl Matrigel (Corning, NY, USA) and subcutaneously injected into fat pads of the mice. Tumor volumes were measured every three days and calculated as follows: width (mm)^2^ × length (mm)/2. All experiments were approved by the Animal Care and Use Committee of Yanbian University.

### Luciferase reporter gene assay

The circMRPS35 and the FOXO1 3′UTR sequences were cloned into the pmirGLO vector to generate wild-type vectors. Vectors containing the mutated sequences were also produced. Cells were seeded in 12-well plates and transfected with either the wild-type or mutated sequence vectors, including the miR-105-5p mimics. The pRL-TK vectors served as an internal reference for normalization. After a 24-h incubation, luciferase activity was measured using the Dual-Luciferase Reporter Assay System (Promega, WI, USA).

### Statistics

Data are presented as means ± SEM of three replicates and were analyzed using GraphPad Prism 7.0 software (CA, USA). The student’s *t*-test or one-way analysis of variance was performed to determine statistical significance between two or more groups. *P*-values < 0.05 were considered statistically significant.

## Supplementary Materials

Supplementary Figure 1

## References

[1] Isakoff MS, Bielack SS, Meltzer P, Gorlick R. Osteosarcoma: Current Treatment and a Collaborative Pathway to Success. J Clin Oncol. 2015; 33:3029–35. 10.1200/JCO.2014.59.4895 26304877 PMC4979196

[2] Harrison DJ, Geller DS, Gill JD, Lewis VO, Gorlick R. Current and future therapeutic approaches for osteosarcoma. Expert Rev Anticancer Ther. 2018; 18:39–50. 10.1080/14737140.2018.1413939 29210294

[3] Cuervo AM. Autophagy: in sickness and in health. Trends Cell Biol. 2004; 14:70–7. 10.1016/j.tcb.2003.12.002 15102438

[4] Eijkelenboom A, Burgering BM. FOXOs: signalling integrators for homeostasis maintenance. Nat Rev Mol Cell Biol. 2013; 14:83–97. 10.1038/nrm3507 23325358

[5] Yang Y, Zhao Y, Liao W, Yang J, Wu L, Zheng Z, Yu Y, Zhou W, Li L, Feng J, Wang H, Zhu WG. Acetylation of FoxO1 activates Bim expression to induce apoptosis in response to histone deacetylase inhibitor depsipeptide treatment. Neoplasia. 2009; 11:313–24. 10.1593/neo.81358 19308286 PMC2657887

[6] Huang H, Tindall DJ. Dynamic FoxO transcription factors. J Cell Sci. 2007; 120:2479–87. 10.1242/jcs.001222 17646672

[7] Bai Y, Chen Y, Chen X, Jiang J, Wang X, Wang L, Wang J, Zhang J, Gao L. Trichostatin A activates FOXO1 and induces autophagy in osteosarcoma. Arch Med Sci. 2019; 15:204–13. 10.5114/aoms.2018.73860 30697272 PMC6348367

[8] Xu R, Liu S, Chen H, Lao L. MicroRNA-30a downregulation contributes to chemoresistance of osteosarcoma cells through activating Beclin-1-mediated autophagy. Oncol Rep. 2016; 35:1757–63. 10.3892/or.2015.4497 26708607

[9] Chang Z, Huo L, Li K, Wu Y, Hu Z. Blocked autophagy by miR-101 enhances osteosarcoma cell chemosensitivity in vitro. ScientificWorldJournal. 2014; 2014:794756. 10.1155/2014/794756 25013866 PMC4072053

[10] Salzman J, Gawad C, Wang PL, Lacayo N, Brown PO. Circular RNAs are the predominant transcript isoform from hundreds of human genes in diverse cell types. PLoS One. 2012; 7:e30733. 10.1371/journal.pone.0030733 22319583 PMC3270023

[11] Vo JN, Cieslik M, Zhang Y, Shukla S, Xiao L, Zhang Y, Wu YM, Dhanasekaran SM, Engelke CG, Cao X, Robinson DR, Nesvizhskii AI, Chinnaiyan AM. The Landscape of Circular RNA in Cancer. Cell. 2019; 176:869–81.e13. 10.1016/j.cell.2018.12.021 30735636 PMC6601354

[12] Xi Y, Fowdur M, Liu Y, Wu H, He M, Zhao J. Differential expression and bioinformatics analysis of circRNA in osteosarcoma. Biosci Rep. 2019; 39:BSR20181514. 10.1042/BSR20181514 31036604 PMC6522716

[13] Shen S, Yao T, Xu Y, Zhang D, Fan S, Ma J. CircECE1 activates energy metabolism in osteosarcoma by stabilizing c-Myc. Mol Cancer. 2020; 19:151. 10.1186/s12943-020-01269-4 33106166 PMC7586679

[14] Zhang Y, Xue W, Li X, Zhang J, Chen S, Zhang JL, Yang L, Chen LL. The Biogenesis of Nascent Circular RNAs. Cell Rep. 2016; 15:611–24. 10.1016/j.celrep.2016.03.058 27068474

[15] Wu Y, Xie Z, Chen J, Chen J, Ni W, Ma Y, Huang K, Wang G, Wang J, Ma J, Shen S, Fan S. Circular RNA circTADA2A promotes osteosarcoma progression and metastasis by sponging miR-203a-3p and regulating CREB3 expression. Mol Cancer. 2019; 18:73. 10.1186/s12943-019-1007-1 30940151 PMC6444890

[16] Jie M, Wu Y, Gao M, Li X, Liu C, Ouyang Q, Tang Q, Shan C, Lv Y, Zhang K, Dai Q, Chen Y, Zeng S, et al. CircMRPS35 suppresses gastric cancer progression via recruiting KAT7 to govern histone modification. Mol Cancer. 2020; 19:56. 10.1186/s12943-020-01160-2 32164722 PMC7066857

[17] Ding S, Zhang G, Gao Y, Chen S, Cao C. Circular RNA hsa_circ_0005909 modulates osteosarcoma progression via the miR-936/HMGB1 axis. Cancer Cell Int. 2020; 20:305. 10.1186/s12935-020-01399-1 32684842 PMC7359231

[18] Fang Y, Long F. Circular RNA circ_0000337 contributes to osteosarcoma via the miR-4458/BACH1 pathway. Cancer Biomark. 2020; 28:411–9. 10.3233/CBM-190647 32390598 PMC12662375

[19] Gu R, Li X, Yan X, Feng Z, Hu A. Circular RNA circ_0032462 Enhances Osteosarcoma Cell Progression by Promoting KIF3B Expression. Technol Cancer Res Treat. 2020; 19:1533033820943217. 10.1177/1533033820943217 33153390 PMC7658513

[20] Wang F, Zhao QH, Liu JZ, Kong DL. MiRNA-188-5p alleviates the progression of osteosarcoma via target degrading CCNT2. Eur Rev Med Pharmacol Sci. 2020; 24:29–35. 10.26355/eurrev_202001_19892 31957815

[21] Yu W, Chen PB, Chen FC, Ding SL, Pan XY. MicroRNA-744 promotes proliferation of osteosarcoma cells by targeting PTEN. Mol Med Rep. 2020; 21:2276–82. 10.3892/mmr.2020.11030 32186762

[22] Cai WT, Guan P, Lin MX, Fu B, Wu B, Wu J. MiRNA-206 suppresses the metastasis of osteosarcoma via targeting Notch3. J Biol Regul Homeost Agents. 2020; 34:775–83. 10.23812/20-72-A-26 32627519

[23] Zheng S, Jiang F, Ge D, Tang J, Chen H, Yang J, Yao Y, Yan J, Qiu J, Yin Z, Ni Y, Zhao L, Chen X, et al. LncRNA SNHG3/miRNA-151a-3p/RAB22A axis regulates invasion and migration of osteosarcoma. Biomed Pharmacother. 2019; 112:108695. 10.1016/j.biopha.2019.108695 30797154

[24] Guan H, Tan P, Xie L, Mi B, Fang Z, Li J, Yue J, Liao H, Li F. FOXO1 inhibits osteosarcoma oncogenesis via Wnt/β-catenin pathway suppression. Oncogenesis. 2015; 4:e166. 10.1038/oncsis.2015.25 26344693 PMC4767937

