# Supplementary material for: Circular RNA circMRPS35 represses malignant progression in osteosarcoma cells via targeting miR-105-5p/FOXO1
Source: Aging (Albany NY). 2024 Aug 5;16(15):11568–76. doi: 10.18632/aging.206022 (PMC11346788; doi:10.18632/aging.206022)
Supplement: Supplementary Figure 1 [file aging-16-206022-s001.pdf]

SUPPLEMENTARY FIGURE

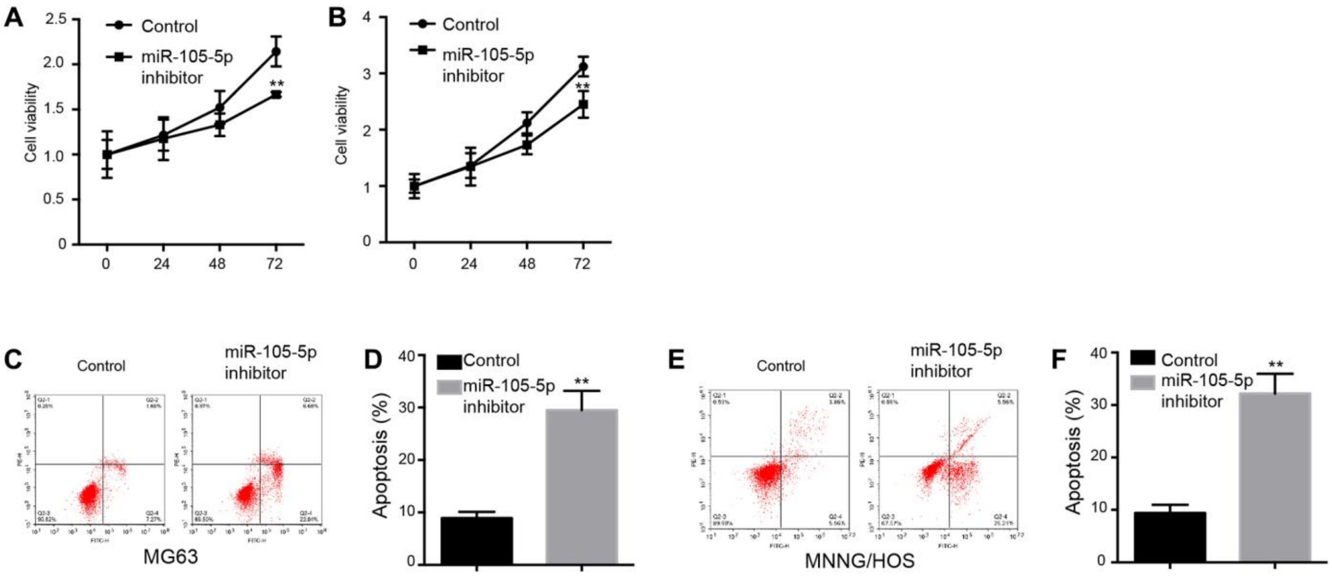

Supplementary Figure 1. The inhibition of miR-105-5p reduces viability and induces apoptosis of osteosarcoma cells. (**A**–**F**) The MG63 and MNNG/HOS cells were treated with miR-105-5p inhibitor. (**A**, **B**) The cell viability was measured by CCK-8 assay. (**C**–**F**) The cell apoptosis was analyzed by flow cytometry analysis. $^{**}P < 0.01$.
